# Improved Free-Energy Estimates for the Permeation of Bulky Antibiotic Molecules through Porin Channels Using Temperature-Accelerated Sliced Sampling

**DOI:** 10.1021/acs.jctc.4c01679

**Published:** 2025-03-12

**Authors:** Abhishek Acharya, Ulrich Kleinekathöfer

**Affiliations:** School of Science, Constructor University, Campus Ring 1, 28759 Bremen, Germany

## Abstract

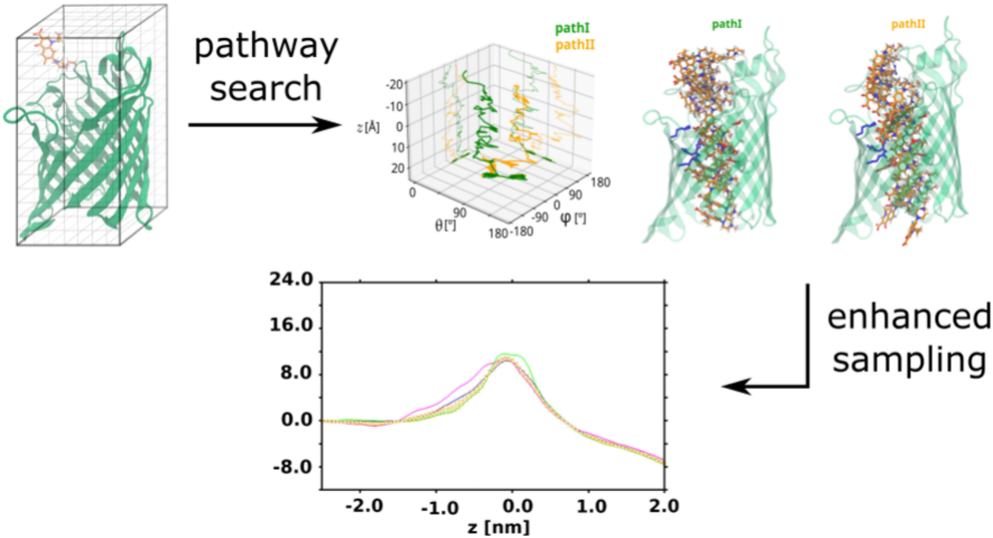

The estimation of accurate free energies for antibiotic permeation via the bacterial outer-membrane porins has proven to be challenging. Atomistic simulations of the process suffer from sampling issues that are typical of systems with complex and slow dynamics, even with the application of advanced sampling methods. Ultimately, the objective is to obtain accurate potential of mean force (PMF) for a large set of antibiotics and to predict permeation rates. Therefore, the computational expense becomes an important criterion as well. Simulation studies on the permeation process and similar complex processes have shown that both the sampling scheme employed and the procedure used for the generation of the initial states can critically affect the quality of the estimates obtained and the respective computational overhead. The temperature-accelerated sliced sampling method (TASS) has been shown to partly address the issues with efficient sampling of the important and slow degrees of freedom by enabling simultaneous biasing of a large number of collective variables. In this work, we investigate the effect of the procedure used for the generation of input conformations on the convergence of free-energy estimates obtained from TASS simulations. In particular, we compare the steered molecular dynamics (MD)-based procedure that has been used in previous TASS studies with the Monte Carlo pathway search method, which is used to obtain approximate permeation trajectories with minimum perturbation of the protein channel. We tested different input setups for enrofloxacin permeation through the porins OmpK35 and OmpE35. The best setup shows an improved agreement between independent PMFs in both cases at a much lower computational cost.

## 1 Introduction

**Figure 1:**
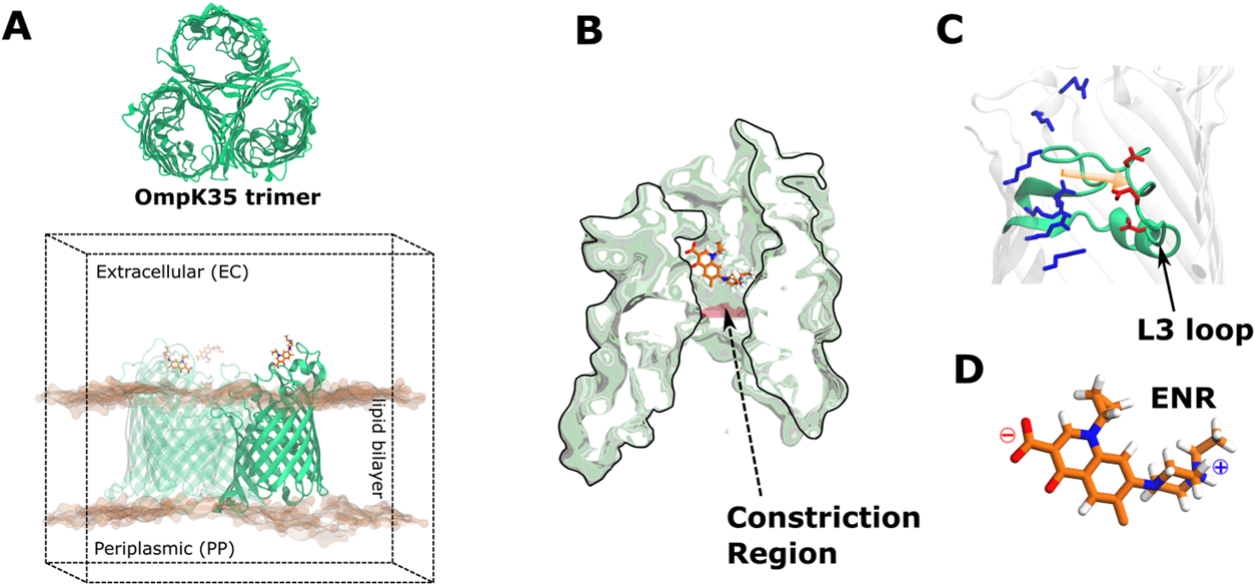
(A) Schematic showing the simulation system used for investigating the antibiotic permeation through OmpF and its orthologues. The trimeric OmpK35 pore is depicted here in green. Also shown is the simulation box with the trimer and one of the monomers being emphasized using a darker green color. The trimeric channel is embedded within a POPE bilayer inside a box of solvent and ions (not shown). (B) Pore lumen on OmpF and related orthologues has an hourglass shape with a constriction region (CR) that acts as a partially selective filter. (C) CR is formed by the L3 loop folded inward toward the lumen of the channel. The acidic (red) and basic (blue) residues at the opposite walls of the CR give rise to an internal electric field indicated by an orange arrow. (D) Zwitterionic enrofloxacin molecule is known to permeate through the OmpF and related porins.

The development of new antibiotics against Gram-negative bacterial pathogens is necessary for tackling the growing threat of antibiotic resistance, as has also been recently reported by the World Health Organization (WHO).^1^ The updated list of bacterial priority pathogens includes additional pathogens in the critical and high-priority groups.^1^ While research efforts addressing the need for novel drugs have led to the development of several novel antibacterial molecules, these have been considered insufficient in tackling the increase of resistant pathogens.^2^ Apart from discovering novel routes of antibacterial action, efforts have also focused on improving the accumulation of various classes of antibacterial candidates. In this direction, experimental studies combined with simulations have investigated factors that may influence the permeation rate across the Gram-negative bacterial outer membrane that acts as an impenetrable barrier to polar molecules.^3−16^ β-barrel proteins in the bacterial outer-membrane channels called porins play a key role in the passage of polar solutes by providing a polar water-filled channel. The OmpF pore and its orthologues have been extensively studied for their role in antibiotic translocation across the Gram-negative outer membrane.^12,15,17,18^ These porins exist as trimers and are composed of a β-barrel structure that forms the polar lumen (Figure 1). Each monomer has an hourglass-like structure with a narrow constriction region (CR) that acts as a partial filter against environmental solutes. The CR forms due to the inward folding of the L3 loop of the barrel and has a characteristic arrangement of charged residues of opposite polarities, leading to a strong internal electric field. This electric field reorients solute molecules with an internal dipole at the CR.

The molecular mechanism of the permeation of antibiotics and also their efflux^19^ has been studied using atomistic molecular dynamics simulations. As the permeation events occur, however, on the time scale of micro- to milliseconds,^20^ typical unbiased simulations for capturing the permeation process are impractical. Only for small antibiotic molecules, actual translocation events can be seen in applied field simulations.^21^ Therefore, investigations have employed various enhanced sampling schemes to accelerate the process. In general, these approaches allow the system to access high-energy states and accelerate rare transitions in practically affordable simulation time scales.^22−30^ Enhanced sampling methods such as the metadynamics approach in its various flavors^24,31−35^ and umbrella sampling (US)^21−23^ have mainly been employed to study solute permeation across membrane channels. In particular, Ceccarelli and co-workers in their early studies used several nanoseconds-long metadynamics simulations to characterize antibiotic permeation pathways for various combinations of antibiotics and porins corresponding to the first barrier-crossing event.^8,10,36−39^ Combined with experimental methods, these and many other studies led to an evolved understanding of the factors that govern solute permeation through porins.^15^ Subsequent studies employed significantly longer well-tempered metadynamics (WTmetaD) simulations for estimating the FES for antibiotic permeation across OmpF^21,40^ and OmpC.^41,42^ The US method^43−45^ and adaptive biasing force (ABF) have also been employed to study the permeation of smaller solutes^46−49^ through bacterial outer-membrane channels. Nevertheless, accurate estimation of free energy for the permeation of bulky antibiotics remains a challenge.

Issues with efficient sampling and large errors in FESs have been reported in the studies of antibiotic permeation through OmpC channel,^41,42^ and were later systematically investigated through the evaluation of US and WTmetaD simulations for the translocation of solutes of varying sizes, i.e., a chloride ion, a monophosphate ion, and a fosmidomycin antibiotic, through the channel OprO.^45^ It was demonstrated that the sampling complexity increases with the size of the solute, affecting the convergence in free energy from independent simulations even with the application bias potential for accelerated sampling.^45^ Foremost, all biased sampling schemes run the risk of introducing such structural artifacts that may contribute to sampling error and obtained free-energy estimates. Part of the problem, at least for collective variable-based sampling schemes employed in these studies stem from the choice of the collective variables (CVs) employed.^50,51^ CVs are essentially functions of the atomic coordinates that can describe the collective motions underlying a process over long time scales and resolve the important metastable states involved. Typically, one or two CVs are used, which are intuitively selected to be the most important ones governing the process of interest. The most commonly used CVs are geometric CVs such as distances, torsions, and root-mean-square deviations (RMSDs) preferred due to their simplicity and interpretability. However, for many complex systems, additional orthogonal degrees of freedom (DOFs) with multiple free-energy basins separated by significant barriers could be important for the overall process. Neglecting these DOFs significantly reduces the sampling efficiency, leading to slow convergence and large errors in calculating the properties of interest. Efforts toward addressing the issue have sought to develop sampling methods that could simultaneously handle a larger number of CVs without much computational overhead, for instance, temperature-accelerated molecular dynamics (TAMD),^52,53^ bias-exchange metadynamics,^54^ parallel bias metadynamics,^55^ and temperature-accelerated sliced sampling (TASS).^56^ Alternative approaches aim at obtaining a complex definition for a CV that captures all of the essential slow DOFs governing a process by either a linear or nonlinear combination of simpler functions^57−61^ or by a low-dimensional representation learned by using artificial neural networks.^62−65^

Biased simulations of antibiotic permeation through nanopores suffer from sampling issues, partly due to the lack of a suitable CV that captures both the solute and the protein dynamics. Typically, the bias is applied to the distance between the center of mass (COM) of the antibiotic molecule and the channel. However, other important degrees of freedom, such as the rigid-body rotation of the solute and its translation perpendicular to the channel axis, internal torsions of the solute, and protein motions, are not explicitly sampled. In the case of the translocation of ciprofloxacin through the porin OmpF, the limitation was resolved by employing the TASS sampling scheme, which enables the inclusion of additional CVs.^66^ The results demonstrated significantly improved sampling and convergence of free-energy estimates when comparing independent TASS simulations on the three OmpF monomers. Due to the advantages of a controlled sampling along the channel axis with acceleration over additional CVs with a minimal computational overhead, the strategy has since become a method of choice, especially for the study of antibiotic permeation process through various OmpF orthologues.^67−70^ Although important mechanistic details have been obtained from these studies, the convergence of free-energy estimates for some other systems of interest using the TASS sampling scheme has proven difficult, with a high computational burden for obtaining convergence. In the present work, we focus on a couple of such cases and explore strategies for further improvements in the convergence of FES estimates and associated computational cost. The methodology for the generation of the initial states for running biased simulations can drastically affect the sampling and the free-energy estimates, particularly in complex systems governed by several slow DOFs.^71,72^ Initial states can be generated via various strategies such as steered MD (SMD), metadynamics, or other coarse schemes such as TAMD. For the study of antibiotic permeation, steered MD has been used to generate the initial configurations that lie along a possible permeation path, which are subsequently used as seeds for enhanced sampling simulations. However, the method can potentially introduce artifacts through drastic perturbation of the protein conformation. In previous studies, we have tried to mitigate the problem by a more careful generation of initial configurations through a stepwise generation procedure and close inspection of the pore structure at each step.^66^ In another study, position restraints have been used to avoid significant structural changes in the pore during SMD runs to generate input configurations.^72^ Recently, Tajkhorshid and co-workers suggested the Monte Carlo pathway search (MCPS) to exhaustively sample the high-dimensional configuration space for the antibiotic molecule inside the OmpF porin and to obtain an optimal permeation pathway that they used to generate initial configurations for one-dimensional bias-exchange US simulations (1D-BEUS).^71^ The method involves the generation of a large set of possible orientations of the antibiotics on a three-dimensional (3D) grid inside the pore and the calculation of the corresponding pore–antibiotic interaction energy. This information is then used to determine a large set of energetically feasible permeation pathways by using a Monte Carlo search scheme. This approach presents a key advantage as it is now possible to select from this set of pathways the prominent paths based on energetics, geometric criteria, or both. In the study by Haloi et al.,^71^ the authors obtained the most likely pathway using Dijkstra’s algorithm. The top pathway was used as input for FES calculations using 1D-BEUS. In the present work, we employ the MCPS approach to generate the initial seed configurations for the TASS simulations. While the original implementation of the procedure uses a host of Tcl and Python scripts for setting up and running the calculations, we use and make available an implementation written entirely in Python that largely automates the process of generating and setting up the inputs for TASS simulation windows. Here, we investigate the permeation of enrofloxacin (ENR) through porins OmpK35 and OmpE35 and compare the performance of the SMD-based TASS approach with that of the MCPS-based TASS runs primarily in terms of convergence from independent biased runs and the computational overhead. The simulations of the ENR permeation through porins has proven to be particularly numerically expensive and hard to converge, as also observed in a previous investigation.^69^ The results show that for the test cases, the MCPS-based approach in general shows an improved convergence between the free-energy estimates obtained for the three monomers at a lower computational expense. At the same time, our tests show that the best convergence is achieved when the three independent biases are applied to the monomers in completely separate runs, as opposed to the strategy of applying three biases to the three monomers within the same pore as have been employed in previous studies using well-tempered metadynamics^41,42^ and TASS.^66,68−70^ Overall, a good convergence of FES estimates from independent simulations is achieved through the combination of a strategy for the generation of initial states that minimally perturb the pore structure, the selection of initial configurations along one of the energetically feasible permeation pathways found by MCPS, and running separate biased simulations on each monomer to obtain the corresponding FES.

## 2 Methods

### 2.1 System Setup for OmpE35 and OmpK35

The simulation systems for this work were taken from a previous study.^70^ Briefly, the coordinates for the orthologues of the OmpF plasmids OmpK35 (*Klebsiella pneumoniae*) (PDB ID: 5O77) and OmpE35 (*Enterobacter cloacae*) (PDB ID: 6ENE) were obtained from the Protein Data Bank. The channel trimer was embedded into a box containing lipids, water, and ions using the Membrane Builder module available in the CHARMM-GUI server.^73,74^ Standard protonation states at pH 7.0 were used for all ionizable side chains except the residues E102 and E110 in OmpK35 and the residue D285 in OmpE35 based on a previous study.^70^ A 1-palmitoyl-2 oleoyl-*sn*-glycero-3-phosphoethanolamine (POPE) bilayer and the TIP3P water model were employed in the simulations (details in Table S1). All simulations were performed using the CHARMM36 force field.^75,76^ Moreover, a parallel LINCS algorithm was used to constrain all of the bonds.^77^ The short-range electrostatics and the van der Waals interactions were calculated using a cutoff value of 12 Å and a switching distance of 10 Å, while the long-range electrostatics were treated using the particle-mesh Ewald approach with a standard grid spacing of 1.0 Å.^78^ The systems were minimized using the steepest descent algorithm and equilibrated in several steps for a total simulation time of 50 ns. Production simulations were performed in the NPT ensemble with the temperature maintained at 300 K using the Nosé–Hoover thermostat with a coupling constant of 1.0 ps^–1^, while the pressure was controlled using the semi-isotropic Parrinello–Rahman barostat at 1.0 bar. In addition, we have used a virtual site setup^79−81^ that enables using a 5 fs time step for integrating the equations of motion. The simulations were performed using GROMACS 2019^82^ patched with PLUMED plugin version 2.4.^83^ The force field parameters for the ENR molecule were obtained from a previous study.^42^ VMD^84^ and UCSF Chimera^85^ were employed to analyze the data and to produce images for this work.

### 2.2 Setup and Procedure for TASS Simulations

**Figure 2:**
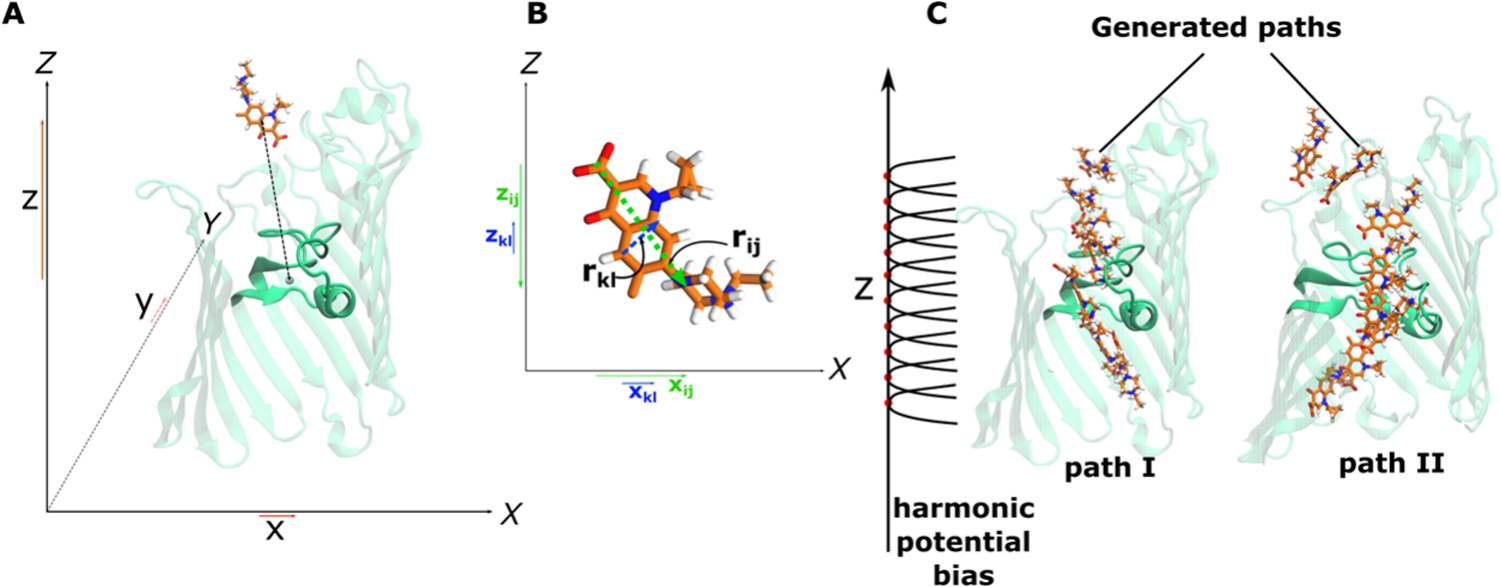
Biased simulations using TASS allow the inclusion of multiple collective variables (CVs) within the sampling scheme. For antibiotic permeation, we bias the CVs describing antibiotic (A) translation and (B) rotation within the channel (see Section 2 for details). (C) The principal CV is *z* that is sampled using a series of harmonic potentials. The input configurations are generated using steered MD (SMD). Two different SMD runs are employed for generating the configurations for path I and path II.

The TASS sampling scheme involves the application of a series of harmonic bias potentials along the principal CV of interest. The method additionally employs the temperature-accelerated molecular dynamics approach (dAFED/TAMD) to accelerate the sampling along the orthogonal CVs.^56^ Thus, the principal CV is sampled through stratification, wherein a large set of simulations is used to apply biases and to restrain the sampling at different positions along the principal CV. Within each simulation window, the additional CVs are sampled by using an elevated temperature. The TASS scheme employs the following Hamiltonian for a system with positions ***R*** and momenta ***P***
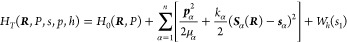
1Here, *H*_0_ denotes the Hamiltonian of the real system that is coupled to a thermal bath maintained at temperature *T*. The *n* auxiliary CVs ***s***_α_ are introduced with momenta ***p***_α_ and respective masses μ_α_. For temperature acceleration, the auxiliary variables ***s***_α_ are coupled tightly to the real CVs ***S***_α_ with force constants *k*_α_. These auxiliary variables are coupled to a thermal bath at higher temperature *T̃*. The term *W*_*h*_(*s*_1_) denotes the harmonic potential bias applied along fictitious CV ***s***_1_. The temperature for the extended space thermostat β̃ is chosen such that the auxiliary variable can drive the real system over the energy barriers that might be encountered during sampling in the real space. Moreover, the parameters *k*_α_ and μ_α_ are optimized for different types of CVs to ensure adiabatic decoupling. In the present study, we apply the harmonic bias potentials along the CV *z* in the range from −2.6 to 2.0 nm. Here, *z* is the CV that coarsely describes the permeation of the antibiotic molecule through the channel. By definition, it is the projection of the COM distance between the antibiotic and the pore along the *Z*-axis (see Figure 2A). This projection is chosen because the pore is by construction aligned such that the long axis of the pore is parallel to the *Z*-axis. In practice, for calculating the COM of the antibiotic and the pore, only the C-α atoms that are part of the relatively rigid β-barrel backbone are used to avoid possible errors in the calculation of the CVs due to structural deviations during biased sampling. The input configurations for each simulation window are generated stepwise, wherein we start with the antibiotic equilibrated for 10 ns at the channel opening on the extracellular (EC) side at position *z* = −2.4 nm. The final configuration from the equilibration at this position is then used as the input for the equilibration at the next window position, i.e., in the present case, at the position *z* = −2.3 nm. The process is repeated to generate configurations for all windows. In practice, it is also necessary to check for any unusual antibiotic or pore conformations. Moreover, significant structural perturbations in the constriction loop and in the side-chain rotations need to be analyzed at the end of each equilibration step. This extra scrutiny is needed to avoid simulation artifacts at the start of the sampling procedure, which itself might lead to sampling errors. Due to the small pore size in the CR, the rotation of bulky antibiotic molecules such as ENR is restricted. Two orientations are possible while crossing the channel from the EC to the periplasmic (PP) side, i.e., with the amino group ahead (orientation I) or the carboxyl group ahead (orientation II). Therefore, for a proper sampling of both orientations, the sampling in the CR (*z* ∈ [−1.0, 0.5] nm) is performed twice with separate umbrellas, i.e., one for each orientation of the ENR molecule. The initial solute–pore configurations for these separate runs in the CR are generated with the respective molecule orientation using the just outlined procedure (Figure 2C). In the next step, the TASS sampling is initiated using harmonic potential biases along the principal CV *z* and temperature acceleration along the orthogonal CVs.^52,53^ The orthogonal CVs include those that describe the rigid-body translation and rotation of the antibiotic molecule within the channel lumen (Figure 2B). Translational CVs are described by the CVs *x*, *y*, and *z* that are projections of connection between the COMs of the antibiotic molecule and the channel along the *X*, *Y* and *Z* axes, respectively. For the rotation, we include the CVs *x*_*ij*_ and *z*_*ij*_ defined as the projections of *r*_*ij*_, i.e., the interatomic vector connecting the carbonyl carbon atom and nitrogen atom of the piperazine ring, along the *X* and *Z* axes, respectively. We also include the CVs *x*_*kl*_ and *z*_*kl*_ that are similarly the projections of the interatomic vector *r*_*kl*_ connecting the C2 and C4 atoms of the quinolone ring that lies approximately orthogonal to vector *r*_*ij*_, as depicted in Figure 2B. In addition, the antibiotic–water interactions are also included within the sampling scheme. For the present simulations, the temperature β̃ of the extended space was set to 900 K using a Langevin thermostat. Moreover, we used a total of 46 windows positioned 0.1 nm apart along the principal CV *z* for sampling all antibiotic orientations. The values of the harmonic force constants range from 2000 kJ/mol/nm^2^ at the channel ends to 6500 kJ mol^–1^nm^2^ in the CR in the case of the OmpK35 channel, and between 2000 and 7000 kJ/mol/nm^2^ for the OmpE35 channel. These values were optimized in short 5 ns equilibration runs to ensure that the histograms along *z* had the expected mean values chosen for the umbrella. An additional 16 windows were used to sample the configurations belonging to path II. As in the previous studies,^41,66^ to reduce the computational cost, we have simultaneously applied TASS biases on the three monomers in a single run to obtain the independent free-energy estimates. Details of the TASS parameters used for the simulations are provided in Section S1.

The TASS method offers several advantages, particularly in the study of antibiotic permeation. Controlled sampling of antibiotic permeation along the pore axis allows for the sampling of specific pathways that are critical for the understanding of the antibiotic permeation. This is especially important for antibiotics that cannot rotate inside the pore due to the narrow pore diameter. In such cases, possible pore orientations must be pregenerated for improved sampling runs. This is more tricky in the case of freely driven sampling approaches such as metadynamics^24,31,32^ and would typically require careful placement of potential walls and postprocessing for correct FES reconstruction. In addition, the incorporation of a large number of CVs allows for a more efficient sampling of antibiotic rigid-body rotations and translations. Even more importantly, the method provides the freedom to select specific CVs for particular windows to enhance the local sampling and improve the overall sampling, as has been demonstrated previously.^66,86^

### 2.3 Setup and Procedure for MCPS Calculations

The setup for MCPS-based calculations requires as input the coordinates of a preequilibrated system with all of the required molecules present in the simulation box, including the antibiotic molecules placed in any arbitrary position inside the box, and an input text file containing various parameters needed for running the calculations. The details of the Python scripts used in this work and their usage are provided on a GitHub page (https://github.com/CPBPG). The details of the MCPS algorithm are provided in a previous study by Tajkhorshid and co-workers.^71^ Briefly, the procedure involves: (1) the generation of orientations of the antibiotic molecule using a Fibonacci spherical lattice,^87^ and translating the sphere over a grid throughout the pore. The configurations with clashes between the antibiotic molecule and the pore are removed. (2) For the remaining configurations, the antibiotic–pore interaction energy is calculated after a short minimization step with appropriate structural restraints on the protein channel. The minimized conformations, their associated *z* position within the pore, inclination and azimuthal angles, and the interaction energies are stored. (3) The MCPS algorithm takes as input the information on the *z* position, inclination and azimuthal angle, and energy values to generate several permeation paths in the three-dimensional conformational space. (4) A filtering step is performed to select and write out energetically feasible pathways and the corresponding coordinates in the form of a trajectory file. (5) Finally, the data of the selected pathway trajectory is used to extract input configurations close to the window positions required by the user for the TASS runs. Each of the above steps together with the relevant parameters and inputs is described in the Section 3, taking the ENR-OmpK35 system as an example.

The scripts implemented in Python offer flexibility in that they enable running MCPS calculations on each individual monomer to generate pathway data sets individually for each monomer. The final setup script can take as input multiple trajectories, one for each monomer, and set up the coordinate files for the TASS windows according to the specifications of the user. While this implementation can generate inputs compatible with the GROMACS simulation engine, the code can be adapted to work with the other simulation software. For the MCPS-based strategy, we first tested the setup and run TASS simulations simultaneously on all three monomers with the antibiotic molecules sampling the same umbrella position in all three monomers. This is the setup that has been used also for previous TASS studies and assumes that the mutual interactions between the antibiotic molecules in the adjacent monomers are minimal due to the shielding effects of the protein channels and the surrounding water molecules. It is with this assumption that we consider the three FES estimates obtained from the setup as “independent”. We term this setup as the *3S* setup. In addition, we also tested TASS simulation setups with the MCPS setup procedure termed *3S-Staggered* and *3D-Independent*. The former involves a staggered positioning of the antibiotic molecules in adjacent monomers, such that different window positions are sampled in the single simulation. The positions are set such that the antibiotic molecules in adjacent monomers are kept at the maximum possible distance from each other to minimize effects due to mutual interactions. In the case of the *3D-Independent* setup, completely independent and separate TASS simulations are performed for each monomer.

### 2.4 Estimation of Free Energy Using TASS and Calculation of the Associated Error

For all simulations, one-dimensional FESs were calculated using the mean force approach as described previously.^66,88^ The average 1D free energy was calculated using a bootstrapping approach based on the histogram bootstrapping method implemented in g_*wham*^89^ and has been used in previous studies on antibiotic permeation.^66−70^ We used 100 bootstrap estimates to calculate the average PMF and the associated error. For obtaining error estimates comparable to those reported in some previous studies, we also calculated the average free energy *F*_avg_(*z*) and error estimates using the following expression (using *M* = 3)
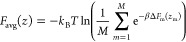
2where *k*_B_ denotes the Boltzmann constant, *T* the temperature, β the inverse temperature expressed as β = (*k*_B_*T*)^−1^, and *ΔF*_m_(*z*_m_) the free energy along the *z* CV for *M* simulations. The error in *F*_avg_(*z*) was determined by standard error propagation.

## 3 Results and Discussion

### 3.1 FES Calculations Using SMD-Based TASS Simulations

**Figure 3:**
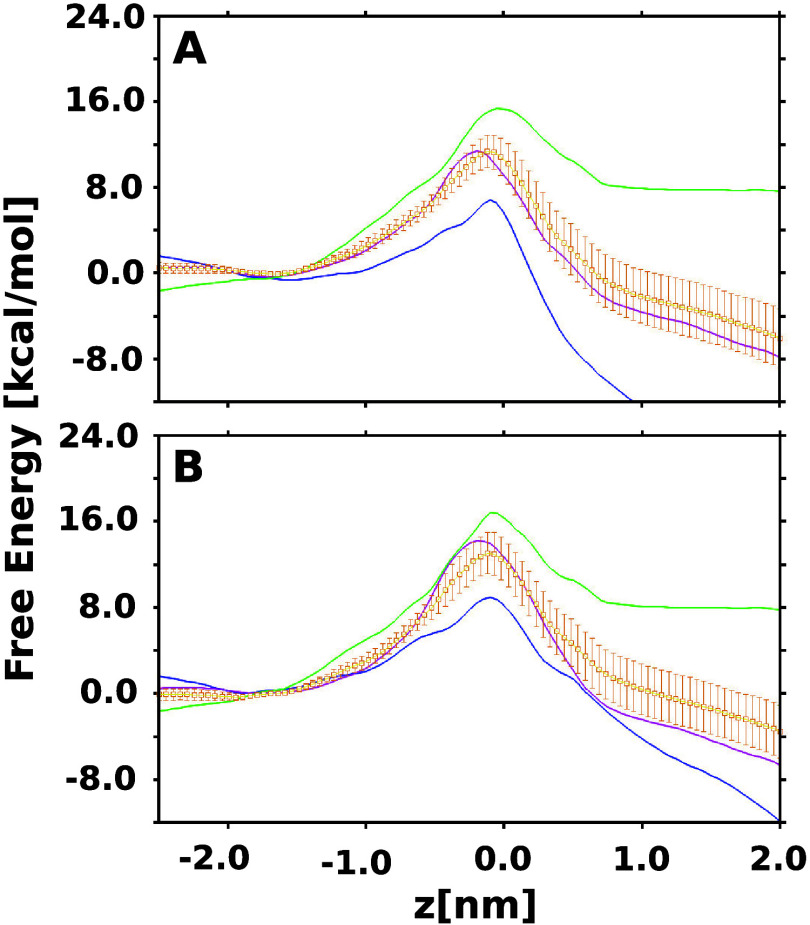
One-dimensional free-energy estimates for enrofloxacin permeation through the OmpK35 channel, calculated by using configurations from SMD-derived paths as inputs for the TASS simulations. The simulations were performed using configurations generated through pulling of the antibiotic molecule from (A) EC to the PP side and from (B) PP to the EC side of the channel. The individual free energies were aligned suitably at a chosen point along *z* to obtain an acceptable fit before the calculation of the error. The average free energy and the associated errors were calculated using the bootstrap approach and are depicted in yellow.

First, we performed TASS simulations for the ENR-OmpK35 system employing the typical setup procedure as used in previous studies^66−70^ and described in the Section 2. Briefly, we sampled ENR configurations during the permeation process along the CV *z*, which is the *Z*-component of the COM distance between the OmpF pore and the antibiotic molecule. The system altogether contains the OmpF trimer embedded in the membrane and three copies of the antibiotic molecule in a box of solvent and neutralizing ions. The harmonic potential biases are thus applied between the three ENR molecules and their respective OmpF monomers to allow sampling for all three monomers in a single run. The input configurations are generated stepwise, starting from a configuration wherein the ENR is positioned at the EC mouth of the OmpF pore. The output of the equilibration at the first window with mean position *z* = −2.4 nm is used as the starting configuration for the equilibration for the next window at *z* = −2.3 nm. Using this procedure, configurations are generated starting at the EC side and ending at the PP side. TASS simulations were performed for a total of about 35 μs. The 1D-FES estimates for the three monomers are shown in Figure 3A. We find large deviations between the free energies of the three monomers. The average FES obtained using the histogram bootstrap approach^89^ provides a barrier height of 10.57 ± 1.46 kcal/mol as shown in Figure S1. For a suitable comparison of the estimates for convergence and error, we previously aligned the individual 1D-FES profiles at a suitable point along the *Z*-axis. Using the fitting, we obtained an average error of 11.39 ± 1.42 kcal/mol (Figure 3A). However, we find a large difference of more than 12 kcal/mol between the lowest and the highest values calculated for the peaks of the three FES. To test for the degree of hysteresis due to the SMD pulling protocol using the umbrella sampling and related methods, it has been suggested previously to carry out enhanced sampling runs from inputs generated using SMD pulling in both forward (EC to PP side) and reverse (PP to EC side) directions.^72^ We therefore repeated the present TASS runs with an SMD-based setup using the initial ENR position at the PP mouth and a generation of configurations in the PP to EC direction. The TASS simulations with the reverse setup produced estimates, shown in Figure 3B, that once again indicate large deviations between the independent PMFs and an average barrier of 13.01 ± 1.41 kcal/mol. Comparison of the “forward” and “reverse” averages suggests some hysteresis effect due to the SMD strategy as delineated in Figure S2. Moreover, the large deviation in the estimated 1D barriers suggests issues with consistent sampling. The source of the inconsistencies in sampling could be due to differences in the initial configurations generated for each of the monomers combined with artifacts due to the SMD pulling and exclusion of the channel degrees of freedom in the sampling scheme. Although in theory the calculated FESs should be independent of the initial path used for the calculations, for complex systems with limited sampling of all of the relevant degrees of freedom, a dependency is observed in practice also in the present study. While using the TASS method, the sampling strategy promotes extensive sampling for the conformations of the antibiotic molecule and antibiotic–solvent interactions; the slow motions along the protein degrees of freedom are still not explicitly included. This omission can potentially lead to inconsistent sampling between independent runs.

### 3.2 Setup and Execution of MCPS Calculations

To address the problems with the SMD-based setup scheme, Haloi et al. suggested a scheme for obtaining the most likely pathway using the Monte Carlo pathway search approach combined with a graph-based search scheme.^71^ They employed antibiotic configurations using this pathway to perform 1D-BEUS simulations of 6-deoxynybomycin (6-DNM) and its derivative. The reasoning behind the approach was to focus sampling efforts on the pathway that captures the relevant slow degrees of freedom during the permeation process. The authors showed that the use of the MCPS-based setup provides FES estimates that are consistent with the experimental data. We also note that the method avoids the application of large external forces during the exhaustive generation of antibiotic configurations inside the pore and performs only an energy minimization of all configurations with restraints on the channel backbone to obtain relaxed conformations. Therefore, we decided to use the MCPS method to obtain the initial pathways for all of the OmpF monomers and used configurations from these pathways as input configurations for the TASS runs.

**Figure 4:**
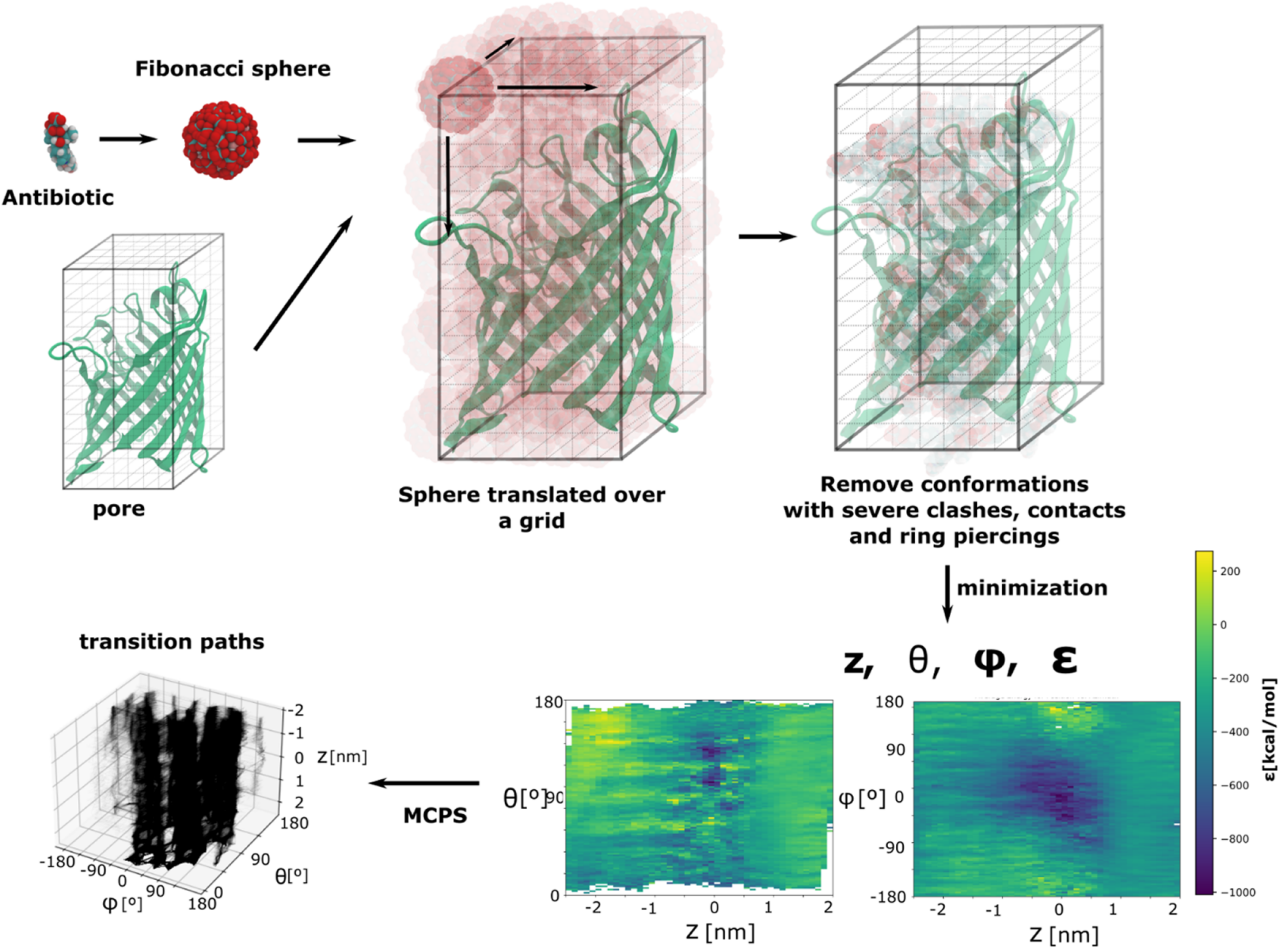
Strategy used for the generation of the MCPS pathways. A Fibonacci sphere of rotational configurations of the antibiotic molecule is translated over a 3D grid covering the pore volume. The data set is cleaned of configurations with clashes, contacts, and ring piercings. The remaining configurations are minimized in the gas phase. For each minimized configuration, the CV *z*, the inclination angle θ, azimuthal angle ϕ, and the protein–antibiotic interaction energy ε are stored. The MCPS algorithm is used to generate the transition paths through the pore. Here, we depict the 5000 trajectories generated for the ENR-OmpK35 system.

For the TASS simulations, we implemented the MCPS setup in Python and enabled simultaneous calculations for all three OmpK35 channels. The procedure is the same as the original approach by Haloi et al., except for a few details that vary (see Figure 4). As input, the code expects a fully equilibrated system consisting of the channel, lipid membrane, antibiotic molecules, water, and ions. Briefly, in the first step, configurations of a solute (drug molecule) within one or more channels of a protein are generated by first rotating a drug to generate points on a Fibonacci sphere followed by translating the sphere on a grid within the pore lumen. This step was performed using a Fibonacci spherical lattice^87^ with 12 points to generate different rigid-body orientations of the antibiotic molecule. We defined a grid of 16 × 16 × 44 with a uniform grid spacing of 0.1 nm. The COM of the respective monomer was used as the reference position for the center of the grid. Copies of the sphere lattice were translated and positioned at all grid points. The antibiotic configurations with clashes and ring piercings were checked for and removed by the script. Ring piercing arises when a protein side chain enters the ring of the antibiotic. These configurations cannot be resolved in minimization and can lead to the system blowing up. This way, we generated a total of 370 K configurations. For each generated configuration, we perform a gas-phase energy minimization, i.e., without water molecules and ions, to relieve existing clashes and obtain relaxed configurations. During this step, we restrain the heavy atoms of all of the channel residues with a force constant of 5000 kJ mol^–1^ nm^–1^, except the side-chain atoms of residues that fall within 0.4 nm of the antibiotic molecule for which restraints with a weaker force constant of 200 kJ mol^–1^ nm^–1^ have been used. This is to avoid large deviations from the equilibrated channel conformations. While Haloi et al. used an implicit solvent during minimization within the NAMD code, the implicit solvent code has been discontinued since GROMACS v2018 due to slow performance and worse accuracy compared to the explicit solvent. Furthermore, since we are interested in only coarse permeation paths and in any case perform minimization with large restraints on the protein atoms, the minimization without water molecules is justified. The minimization step is the most time-consuming stage. Therefore, we divide the total data set into smaller chunks of about 20,000 frames and run the minimization procedure on each chunk simultaneously to accelerate this step. It is important to note here that the script is written to work with GROMACS and requires proper input topology files, restraint files, and run parameter files from the user to perform the minimization step correctly, and to write out the *z* position, the inclination θ and azimuthal ϕ angles, and the antibiotic–channel interaction energy. The θ angle can be calculated from the *Z*-component of the antibiotic vector and the ϕ is defined as the angle between the projection of the antibiotic vector and the internal electric field vector on the *XY*-plane (see Figure S3). User-specified atoms are used by the script to determine the antibiotic vector. For the ENR molecule, we used the C15 and N22 atoms as the head and tail of the vector, describing the orientation and position of the drug molecule. The internal electric field is conveniently defined by specifying one head and one tail residue within the pore. For OmpK35, we used residues ASP113 and ARG74 as the head and tail residues, respectively. The minimized configurations are written out as a PDB file and the corresponding values for *z*, θ, ϕ, and the antibiotic–pore interaction energy ε are stored. Next, the Monte Carlo pathway search algorithm identifies a user-specified number of energetically feasible paths in high-dimensional space. We performed the MCPS step in the *z* – θ – ϕ space of the antibiotic molecule as described previously^71^ to generate energetically feasible pathways. Previous studies on the permeation of bulky antibiotics have shown that due to the narrow CR, the antibiotic molecule has a restricted rotation in the CR and can permeate through the CR in two possible orientations with the antibiotic vector aligned parallel (θ = 0°) or antiparallel (θ = 180°) to the *Z*-axis. To ensure sampling of both configurations, SMD runs were used previously to generate initial configurations for both possible paths. Therefore, we did not perform the graph search step that was used by Haloi et al.^71^ for the selection of the most likely pathway, but directly classified 5000 pathways into path I and path II based on the average inclination angle observed at the CR. To this end, we chose a deviation of ±40° around 0 and 180° for path I and path II, respectively. Finally, the clustered pathways were sorted based on the average interaction energies in the CR and the top pathways from path I and path II cluster were stored. The top-ranked pathway after the sorting step was used for the final setup step. It must be noted here that the top pathway selected from these two clusters is just one of many possible energetically feasible pathways. Visual analysis of the top pathways obtained in this manner revealed no substantial qualitative differences in the antibiotic–pore interaction modes. However, we note a clear difference in terms of the antibiotic orientations between the path I and path II trajectories (discussed in the next section).

For the final preparation of the simulation windows for TASS simulations, the selected trajectory file containing the data for the top transition path is used. Essentially, for each mean position of an umbrella potential, the code searches for the closest node on the given pathway and uses the corresponding structural data to generate the input coordinate file. For the trimeric pore used here, we prepared the system to enable TASS sampling for all three monomers in a single simulation run, also termed the *3S* setup. Simultaneous sampling of antibiotic configurations in the three monomers has been used in previous metadynamics^41,42^ and TASS^66,70^ simulations.

### 3.3 MCPS-Based TASS Simulations for OmpK35

**Figure 5:**
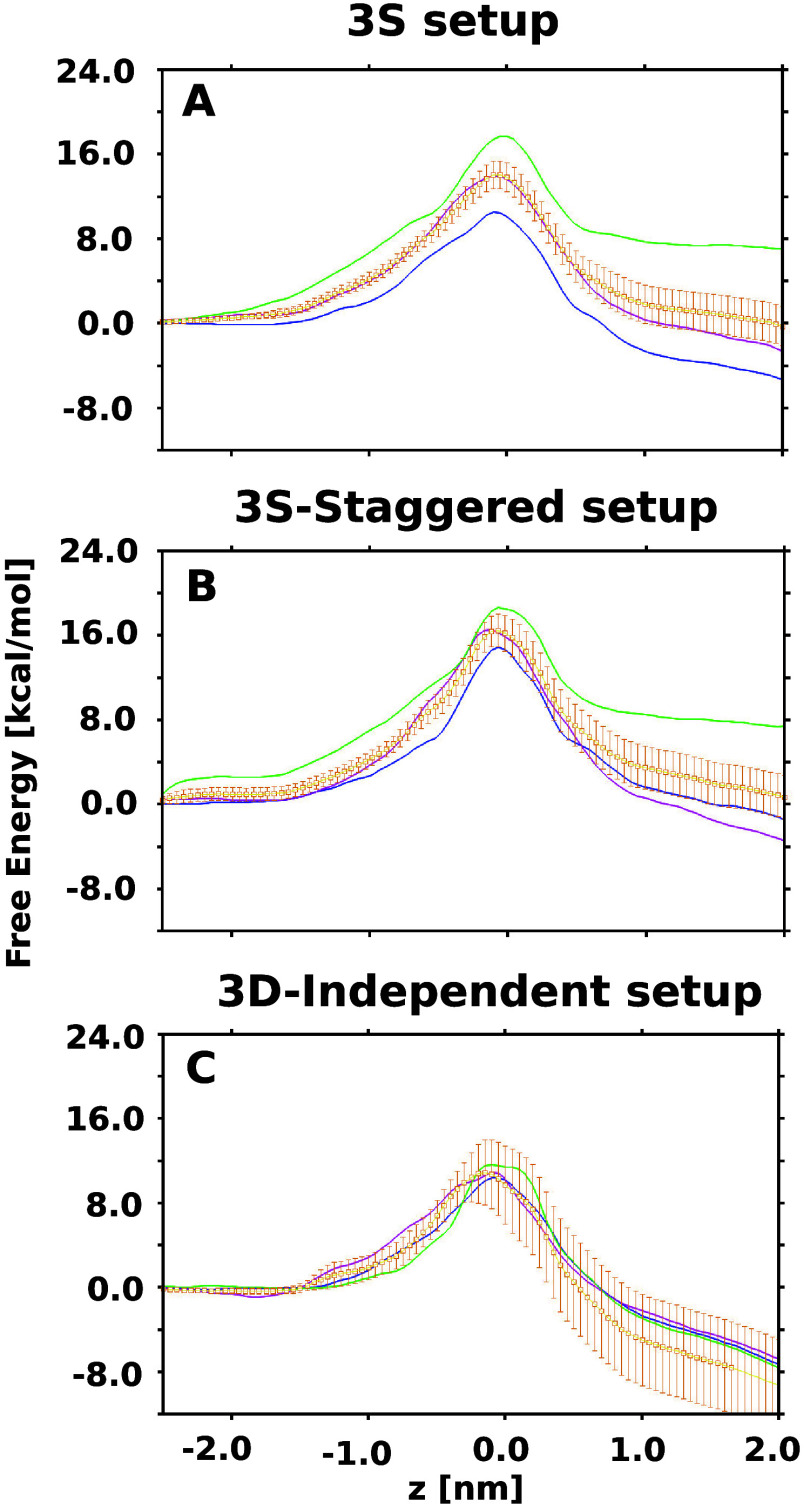
One-dimensional free-energy estimates for enrofloxacin permeation through the OmpK35 channel, calculated by using configurations from MCPS-derived paths as inputs for TASS simulations. Simulations were performed with (A) simultaneous bias on the three monomers with the same umbrella mean, (B) simultaneous bias on the three monomers with a staggered umbrella mean, and (C) bias on the three monomers in separate simulation runs. The average free energies and the associated errors were calculated using the bootstrap approach and depicted in yellow.

The pathways corresponding to path I and path II obtained from the MCPS calculations on the three pores were used to run TASS simulations, as indicated in Figure S4A. The plots for the input trajectories show that the trajectories for path I and path II differ in the inclination angle assumed while crossing the CR region around *z* = 0.0. This is also observed in the adjacent antibiotic configurations for the two paths in Figure S4A. We performed 100–200 ns-long simulations per window, yielding a total of close to 19 μs of sampling. The 1D-FESs in Figure 5A show an improved agreement between the individual FESs for the three monomers, compared to those obtained with the previous SMD-based setup. In this case, a barrier with an average height of 14.39 ± 1.00 kcal/mol is obtained. However, the difference between the individual barriers is still around 8 kcal/mol, a substantial difference. We would like to point out that the energy barriers obtained for the ENR-OmpK35 system thus far is lower than the barrier of 15.40 kcal/mol obtained from the 1D-FES estimates for the ENR-OmpF system. The OmpK35 channel possesses a wider pore with a diameter of 0.72 nm compared to that of OmpF with a diameter of 0.62 nm. This fact has been used earlier to explain the faster diffusion of penicillins through OmpK35 than through OmpF.^17^ More recent extensive investigations suggest, however, that the permeability is also affected by other factors including the electrostatics of the pore and its dynamics, as well as properties of the antibiotic molecule such as charge distribution, size, and dipole moment.^4,5,7,9−11,18,21,40,68,90^ The available permeability data set of a wide set of drugs for different OmpF orthologues suggests that zwitterionic antibiotics with a strong internal dipole moment show a greater permeability through OmpK35 than through OmpF.^18^ Since ENR is also a zwitterionic antibiotic with a large internal dipole moment of around 36 D, it is likely that ENR also shows a faster permeation through OmpK35 compared to OmpF. Previous estimates for ciprofloxacin, an antibiotic similar to ENR, have provided a similar trend with a permeation barrier of about 13.50 kcal/mol through OmpF and 11.69 kcal/mol through OmpK35 from 1D-FES.^66,70^

The issue with the *3S* setup is that in concurrent biased simulations of three antibiotic molecules at the same position along the channel, the dynamics of one antibiotic may affect the other. With the *3S* setup, it was assumed that such effects may be minuscule due to the screening effect of water and the pore itself. In a previous study employing the US method, we employed a staggered placement of antibiotics with the molecular copies in adjacent monomers placed as far as possible in a single simulation.^45^ While the study provided no comparison between the staggered (hereafter termed *3S-Staggered* setup) and the *3S* setup, practically setting up a staggered placement of antibiotics was time-consuming and required much care during input generation, especially with the SMD-based approach. Due to the automated setup of the inputs based on MCPS pathways, it was now possible to implement the *3S-Staggered* setup and perform TASS simulations. Based on the 47 windows used for covering the full range along the *z* CV, the maximum possible displacement was about 15 umbrella means apart. For setting up the *3S-Staggered* inputs, the script takes the required umbrella means series and generates a new series by circular permutation with a shift of 15 positions. This is repeated to generate a third series. These three series of umbrella means are used, respectively, for setting up the staggered antibiotic positions for the three monomers. Figure 5B shows that with a total simulation of 19 μs, we obtain an improved agreement between the individual estimates showing a maximum difference between the independent barrier estimates of around 4 kcal/mol. However, the average free-energy barrier is higher, with a value of 16.49 ± 1.32 kcal/mol.

Finally, we also performed independent TASS simulations with separate runs for each monomer to completely negate the possibility of mutual interactions. This setup was termed the *3D-Independent* setup. While this approach is computationally more expensive with 3 times the computational expense, we wanted to determine if TASS combined with the MCPS scheme provides better results and a good agreement between the independent estimates. All other parameters were identical to those used for the aforementioned simulations, and each set was run for a total of ≈12 μs. With this setup, we obtained the best agreement between the independent barrier estimates with a maximum difference of 1.2 kcal/mol (see Figure 5C). The average barrier was calculated to be 10.91 ± 2.27 kcal/mol. However, the estimated error obtained from the bootstrapping procedure is still large.

Recently, Lapierre and Hub investigated the convergence of 1D-FES for fosmidomycin permeation through the OprO porin estimated using different US approaches and its advanced flavors.^72^ The authors reported errors of less than 1.0 kcal/mol for the FES estimates obtained from independent Replica-Exchange US simulations. The reported errors obtained with the other schemes tested were much greater. The study reports averages and standard errors from the independent runs. For suitable comparison, the average and error estimate were calculated using eq 2 for the MCPS runs with the *3D-Independent* setup to be 10.78 ± 0.35 kcal/mol as shown in Figure S5A. It is also interesting to look at the convergence times for all simulations. As noted in our previous studies,^45,66^ we again find that while the individual simulations appear to be mostly converged, there are large errors when comparing the independent estimates (see Figure S6). Notably, for the MCPS *3D-Independent* setup, convergence of the individual runs is achieved in around 8 μs. Moreover, we find that the deviations among the initial free-energy estimates (*t* = 2 μs) are lower compared to those for the *3S* and the *3S-Staggered* setups, although the initial states used for these runs were identical. This finding also shows that for the ENR-OmpK35 system, a simultaneous bias on all three trimers in a single simulation run leads to large errors, possibly due to the interference from the simulation in adjacent pores. We also note that even with the improvement in the free-energy convergence, significant differences in the energies at the ends of the channel remain. This discrepancy can potentially be due to the exclusion of protein degrees of freedom from the sampling scheme. Although using the MCPS strategy, it is possible to generate input pathways with minimal structural perturbations of the pore, the exclusion of important DOFs during the TASS sampling can still lead to errors. In previous studies, it has been established that conformational changes of the L3 loop play a critical role in the permeation process.^67,71^ The inconsistent sampling of loop conformations in adjacent windows and between simulations in different monomers can lead to errors in independent estimates.

**Figure 6:**
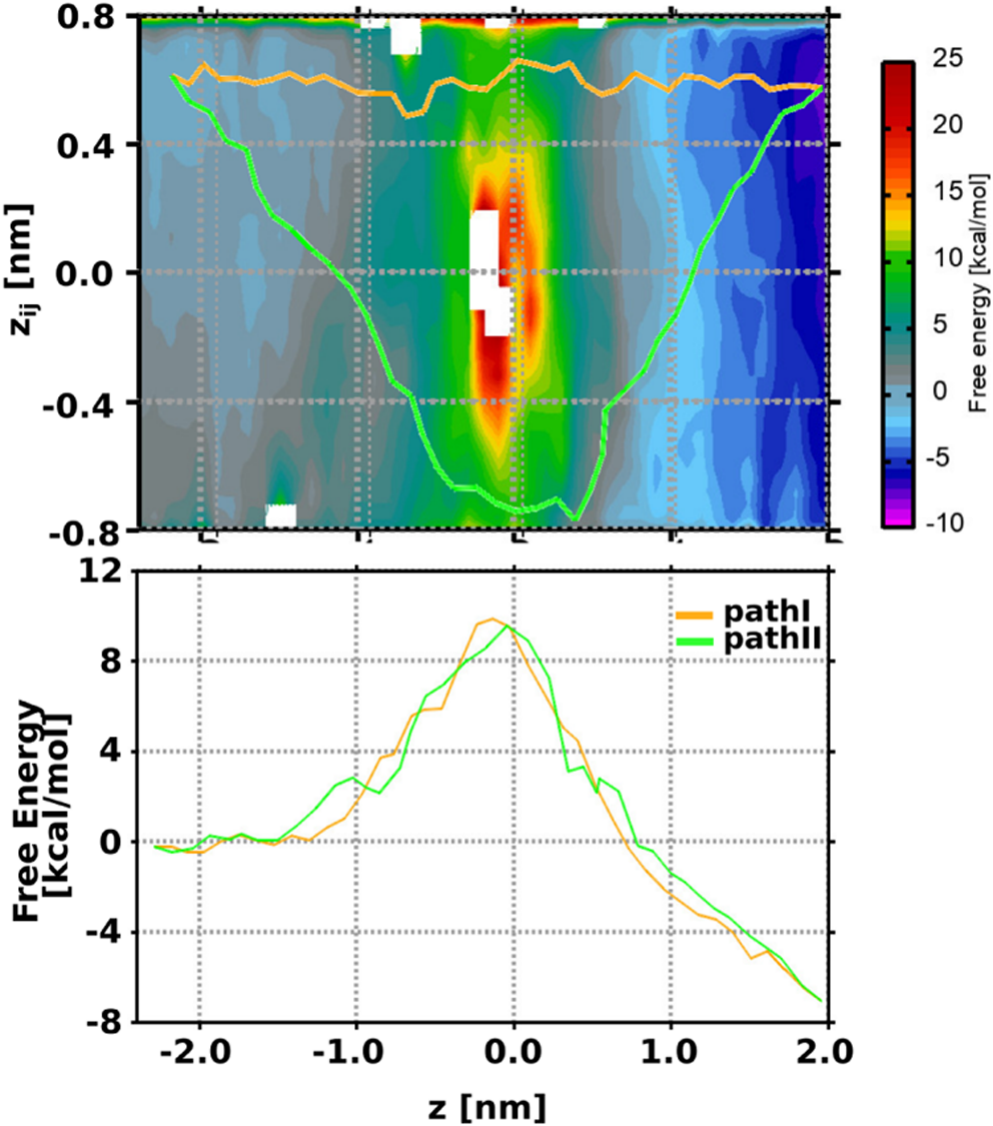
Two-dimensional free-energy estimates for enrofloxacin permeation through the OmpK35 channel obtained from the 3D-Independent MCPS setup. The upper panel shows the free energy within the *z* vs *z*_*ij*_ space. Also shown are the two energetically feasible minimum free-energy paths, path I and path II, obtained using the zero-temperature string method. The bottom panel plots the free energy associated with both permeation paths.

To obtain insights into the sampling and energetics of the two possible permeation paths, we constructed the two-dimensional (2D) FES within the *z* vs *z*_*ij*_ space that represents the positions and orientations of ENR within the channel. The central region has a narrow undersampled region that represents orientations of the ENR that are forbidden due to the narrow CR around *z* = 0. Figure 6A shows that the two paths were adequately sampled. Using the zero-temperature string method,^91^ we also obtained the corresponding minimum free-energy paths (MFEPs). The system encounters similar energy barriers of 10.33 and 10.00 kcal/mol along both path I and path II, respectively.

### 3.4 MCPS-Based TASS Simulations for OmpE35

**Figure 7:**
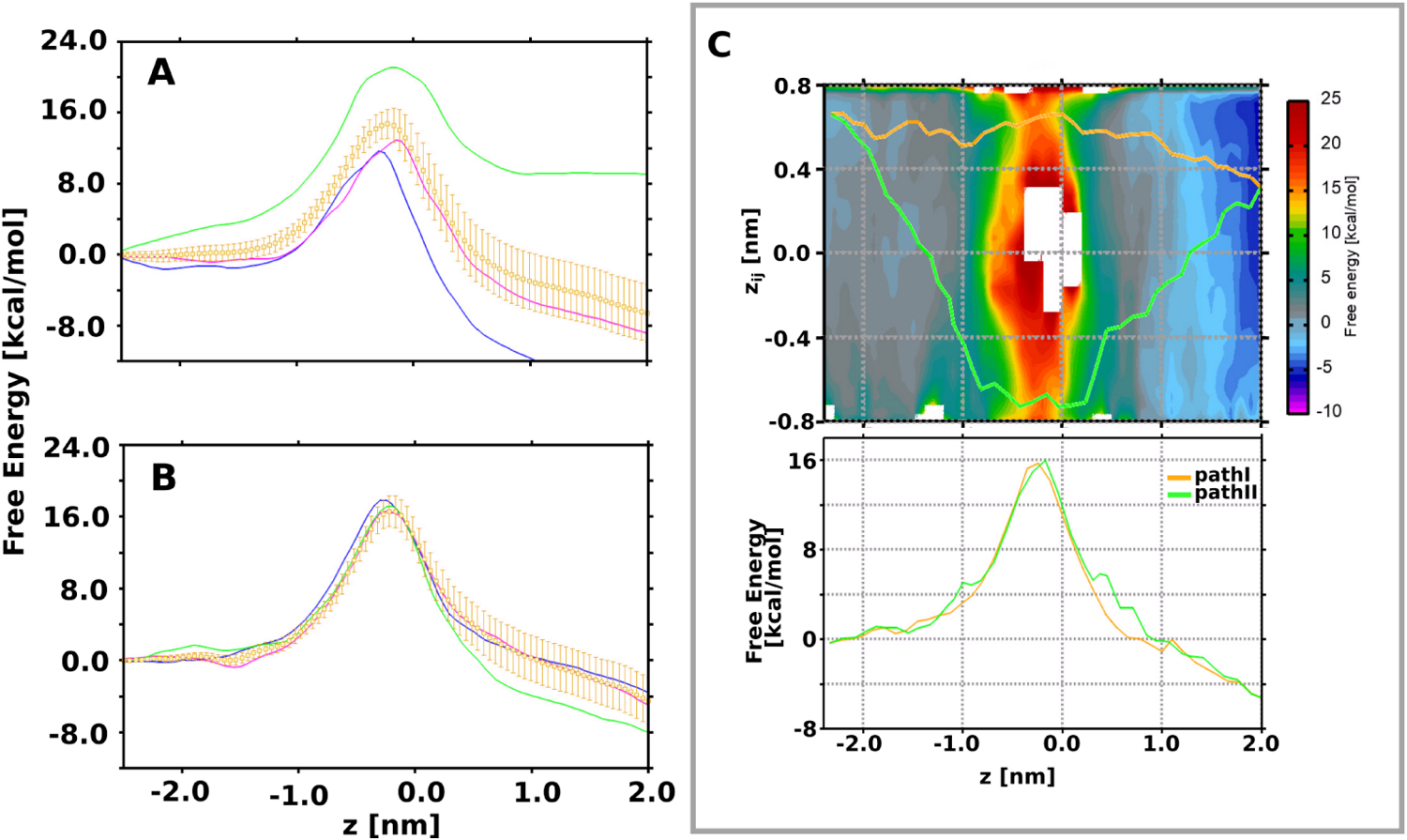
One-dimensional free-energy estimates for enrofloxacin permeation through the OmpE35 channel calculated using configurations extracted from (A) paths generated using SMD and (B) MCPS-derived paths as inputs for the TASS simulations. The average free energy and the associated errors were calculated using the bootstrap method and are depicted in yellow. (C) Two-dimensional free-energy estimates for enrofloxacin permeation through the OmpE35 channel obtained from the 3D-Independent MCPS setup. The upper panel shows the free energy within the *z* vs *z*_*ij*_ space. The two minimum free-energy paths, path I and path II, were calculated using the zero-temperature string method. The bottom panel depicts the free energy associated with both permeation pathways.

Moreover, we tested the MCPS-based TASS simulations on the ENR-OmpE35 system, which also has been shown to be problematic in obtaining consistent sampling and in barrier estimates with the SMD-based TASS runs. The initial runs with the SMD-based TASS setup were performed with a total simulation time of about 30 μs. Figure 7A indicates significant problems with sampling, showing a large difference in the individual barrier estimates of about 10 kcal/mol. The average barrier estimate is 14.60 ± 1.96 kcal/mol. Average and error estimates after suitable alignment of the 1D profiles about a chosen point along *z* give an average barrier of 13.76 ± 1.98 kcal/mol. MCPS calculations were performed with the same parameters that we used for the previous system and obtained path I and II trajectories for each of the three OmpE35 monomers. The paths depicted in Figure S4B show that these two paths are distinct in the inclination angle of ENR at the CR. These pathways were used to prepare inputs for running TASS simulations in the *3D-Independent* setup, which yielded the best results in the previous approach. The 1D-FES obtained from about 19 μs TASS simulations are depicted in Figure 7B. The results show that with this setup of TASS simulations, we obtain good agreement between the individual barrier estimates, with a maximum difference of 1.40 kcal/mol between the different barriers. The average 1D barrier for permeation is estimated to be 16.57 ± 1.50 kcal/mol using the bootstrapping method. The standard error calculated using eq 2 is 16.98 ± 0.58 kcal/mol (Figure S5B). This result provides additional validation for the MCPS-based *3D-Independent* setup. Note that in all error estimates for the MCPS-based TASS simulations on both OmpK35 and OmpE35, we have not performed any alignment of the individual 1D PMFs before the error estimates. The convergence plots in Figure S7 show that the individual plots are once again largely converged. For the MCPS-based TASS run, convergence is achieved after around 12–15 μs of total simulation time. Finally, the 2D FES constructed from the MCPS runs and the calculated MFEPs show that the system encounters similar energy barriers along both path I and path II with values of 16.20 and 16.33 kcal/mol, respectively, as can be seen in Figure 7C. Overall, we see a significantly higher barrier for permeation through OmpE35 than through OmpK35, which can be explained on the basis of the narrower pore size in the former channel.

## 4 Conclusions

Accurate free energies for permeation have been used to estimate the permeability of antibiotics through bacterial porins.^7,18,38,39,92−94^ Practically, the estimation of free energies for bulky antibiotics has proven to be challenging, often with large errors in the resulting estimates from biased simulations. Sampling issues in CV-based approaches arise from the difficulty in selecting CVs that capture all of the important and slow DOFs involved in the permeation process.^26,30,95^ For the investigations on antibiotic permeation, these include antibiotic rigid-body rotation and translation, antibiotic internal DOFs, and protein dynamics.^15,71^ Slow dynamics along one or more of these DOFs can significantly hamper efficient sampling. A related issue is the generation of the seed conformational states for initializing simulations, which has been shown to affect sampling and the resulting free-energy estimates.^71,72^ SMD-based methods involve pulling antibiotics through the channel and generating forces that can introduce structural artifacts. Thus, as the size of the antibiotic increases, both the initial generation of seed states and the sampling of relevant DOFs become more problematic. Previous studies have tried to address the former issue by suggesting recipes that involve a stepwise generation of inputs^66^ or the use of positional restraints on the pore during the SMD step.^72^ Combination of such strategies with advanced sampling schemes has demonstrated improvements in sampling and convergence, notably with errors smaller than 1 kcal/mol using the REUS method.^72^ In this study, we investigated the effect of the initial setup scheme in determining the accuracy of TASS simulations. In particular, the stepwise SMD-based setup that has been used previously shows problems with accurate FES estimation. Using an alternative path search scheme described previously by Haloi et al.,^71^ one can automatically generate coarse permeation paths through the channel, which can be used later for setting up the TASS windows. The method does not involve the use of any artificial forces to obtain antibiotic configurations and thereby avoids introducing structural perturbations and the resulting hysteresis effects. We find that the TASS runs based on MCPS-derived inputs produce better results in terms of agreement between the independent 1D barrier estimates. The automated setup of the windowing scheme used for the TASS runs enables flexibility in setting up multiple parallel TASS simulations for the trimeric porins. Moreover, we find that running completely independent sampling for the three monomers in separate simulation runs provides the best convergence between independent runs and would be the preferred strategy for future work.

A significant advantage of the TASS scheme is the low computational expense for obtaining FES estimates. In previous studies, we have obtained converged free energies with 15–25 μs of simulations for a monomer. In the present work, we also demonstrate that the method can provide converged estimates from 12 μs for ENR-OmpK35 and 19 μs for the ENR-OmpE35 system. The cost of running MCPS is comparatively low, with the energy-minimization step being the computationally demanding procedure. This step can be trivially parallelized for faster processing times. The ease of calculating MCPS-derived paths and the generation of inputs with minimal perturbations to the pore structure appear advantageous for all methods that employ a windowing scheme. This is particularly important in the case of pores that consist of flexible segments that otherwise may be easily perturbed by the forces used in pulling simulations, damaging the pore geometry. Additionally, the MCPS data sets generated as such could be leveraged for setting up virtually any form of calculations that involve running a large set of trajectories, for instance, in milestoning,^96,97^ Markov-state modeling,^98^ and weighted ensemble methods.^99^ Moreover, the reduced computational burden for obtaining converged estimates suggests that running a single TASS simulation for future investigations would be feasible to obtain reliable estimates. Note that in all previous studies, we have performed three simulations to examine the reliability of the estimates for the method of interest, as opposed to single runs accompanied by statistical error estimates.

Sampling issues, however, still persist in the study of antibiotic permeation. It is important to note that detailed studies thus far have involved mostly antibiotic molecules with low molecular mass and rigid structure. Restrictions on the rotation of the antibiotic molecule using potential walls have also been employed to promote the sampling of specific orientations during permeation.^72^ However, for antibiotics with a large internal flexibility, further complications are unavoidable. Efficient sampling would require a way to bias the rotation along the internal dihedral DOFs of the molecule. Furthermore, the generation of the starting seed conformations itself would not be straightforward, with greater chances of generating unproductive antibiotic and channel conformations with the system stuck in deep local minima in a high-dimensional space. This apart, the conformational sampling of the channel, in particular the local fluctuations in the loop segments in the presence of antibiotic molecules itself, presents problems. Methodological developments are needed to address these issues.

From a biological point of view, the improvement in free-energy estimates for antibiotic translocation processes opens up avenues for the investigations of the permeation through pores embedded in more complex membranes. In Gram-negative bacteria, the outer membrane is composed of lipopolysaccharides on the outer leaflet.^100^ Studies have already demonstrated the effect of LPS on channel dynamics and solute translocation energetics.^101,102^ Particularly, simulations indicate that differences in the LPS models considered may affect the structural motions in the external and constriction loop in porins.^103^ In light of the role of loop dynamics during the antibiotic permeation process, it is important to revisit previous simulation studies and models to examine the role of species-specific LPS in loop stabilization and antibiotic-induced dynamics during permeation. Combined with improvements in computational hardware and the recent developments in coarse-grained LPS models that enable improved modeling of protein–LPS interactions,^104^ it will be feasible in the future to perform detailed investigations of more complex membrane models.

## Supporting Information Available

TASS simulation parameters; table of collective variables employed for TASS simulations; one-dimensional free-energy estimates and error; free energies from forward and reverse SMD-based TASS setup; MCPS input paths used for MCPS-based TASS runs; and free-energy convergence plots (PDF)
