# Supplementary material for: Improved Free-Energy Estimates for the Permeation of Bulky Antibiotic Molecules through Porin Channels Using Temperature-Accelerated Sliced Sampling
Source: J Chem Theory Comput. 2025 Mar 12;21(6):3246–59. doi: 10.1021/acs.jctc.4c01679 (PMC11948331; doi:10.1021/acs.jctc.4c01679)
Supplement: Supplementary file 1 — ct4c01679_si_001.pdf [file ct4c01679_si_001.pdf]

Supplementary Information: Improved Free Energy Estimates for the Permeation of Bulky Antibiotic Molecules through Porin Channels using Temperature Accelerated Sliced Sampling

Abhishek Acharya and Ulrich Kleinekathöfer*

School of Science, Constructor University, Campus Ring 1, 28759 Bremen, Germany

E-mail: ukleinekathoefer@constructor.university

S1 TASS parameters

S1.1 OmpK35 simulations

Umbrella sampling parameters:

Restraint positions (nm): -2.5, -2.4, -2.2, -2.0, -1.8, -1.6, -1.5, -1.4, -1.3, -1.2, -1.1, -1.0, -0.9, -0.85, -0.8, -0.75, -0.7, -0.65, -0.6, -0.55, -0.5, -0.45, -0.4, -0.35, -0.3, -0.25, -0.2, -0.15, -0.1, -0.05, 0.0, 0.1, 0.2, 0.3, 0.4, 0.5, 0.6, 0.7, 0.8, 0.9, 1.0, 1.2, 1.4, 1.6, 1.8, 2.0.

κ (kcal/(mol nm^2)) : 478, 478, 478, 478, 478, 598, 598, 598, 598, 598, 717, 717, 836, 836, 956, 956, 1195, 1195, 1314, 1434, 1553, 1553, 1434, 1434, 1434, 1434, 1314, 1075, 956, 956, 956, 836, 717, 717, 597, 597, 597, 597, 478, 478, 478, 478, 478, 478, 478, 478

S1.2 OmpE35 simulations

Umbrella sampling parameters:

Restraint Position (nm): -2.5, -2.4, -2.2, -2.0, -1.8, -1.6, -1.5, -1.4, -1.3, -1.2, -1.1, -1.0, -0.9, -0.85, -0.8, -0.75, -0.7, -0.65, -0.6, -0.55, -0.5, -0.45, -0.4, -0.35, -0.3, -0.25, -0.2, -0.15, -0.1, -0.05, 0.0, 0.1, 0.2, 0.3, 0.4, 0.5, 0.6, 0.7, 0.8, 0.9, 1.0, 1.2, 1.4, 1.6, 1.8, 2.0.

κ (kcal/(mol nm^2)) : 478, 478, 478, 478, 478, 478, 597, 597, 717, 717, 717, 717, 717, 836, 836, 956, 1314, 1434, 1553, 1553, 1673, 1673, 1673, 1553, 1553, 1434, 1314, 1075, 956, 956, 956, 836, 717, 717, 597, 597, 597, 597, 478, 478, 478, 478, 478, 478, 478

The TAMD parameters are provided in Table S1 below.

Table S1: Collective variables included in the sampling scheme for both OmpE35 and OmpK35 simulations.

**CV Type**	**CV definition**	**TAMD parameters**
Solute Translation	COM distance projections on the X, Y and Z axes	$\kappa = 5 \times 10^5$ kJ mol^{-1} nm^2<br>$\tau = 0.50$ ps
Solute Rotation	Interatomic vector projections on the X, Y and Z axes	$\kappa = 2 \times 10^5$ kJ mol^{-1} nm^2<br>$\tau = 0.10$ ps
Solute-water Interaction	Coordination Number	$\kappa = 10^4$ kJ mol^{-1} nm^2<br>$\tau = 0.40$ ps

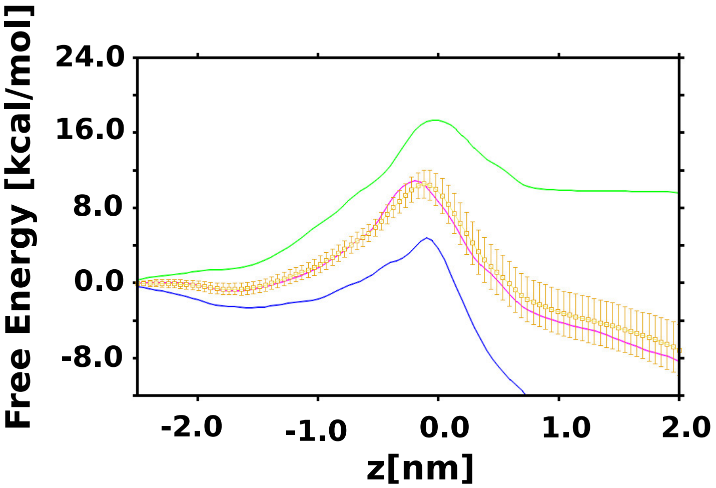

Figure S1: One-dimensional free energy estimates for enrofloxacin permeation through the OmpK35 channel calculated using configurations from SMD-derived paths as inputs for the TASS simulations. The individual free energy profiles were not aligned before the error estimation, as has been done in case of SMD-based TASS simulations.

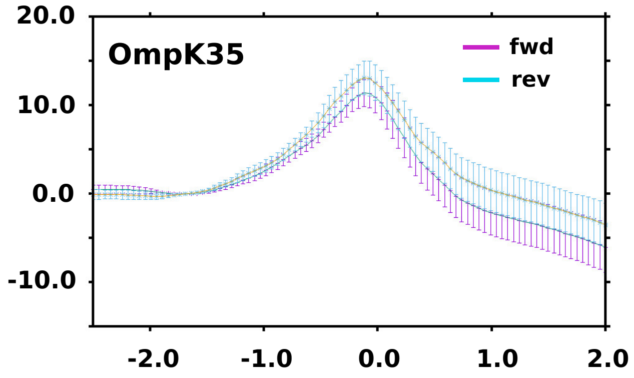

Figure S2: Comparison of the average free energy profiles and their associated errors calculated for the TASS simulations with inputs derived from SMD-based paths. 'fwd' denotes SMD pulling of antibiotic from the EC to the PP side and 'rev' denotes pulling from the PP to the EC side of the channel. The errors have been calculated using the bootstrap approach.

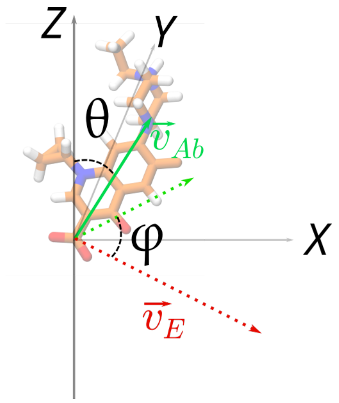

Figure S3: Calculation of the inclination θ and azimuthal ϕ angles used for the MCPS calculations. The inclination of the antibiotic molecule is the angle between the chosen antibiotic vector $\vec{v}_{Ab}$ and the Z-axis. The azimuthal is calculated as the angle between the projection of the vector $\vec{v}_{Ab}$ on the XY plane and the projection of the internal electric field vector $\vec{v}_E$ on the XY plane. The $\vec{v}_{Ab}$ for ENR is calculated using the C15 and N22 atoms as the head and tail of the vector, respectively. The $\vec{v}_E$ vector is calculated using the Cα atoms of Arg74 and Asp113 as head and tail atoms, respectively.

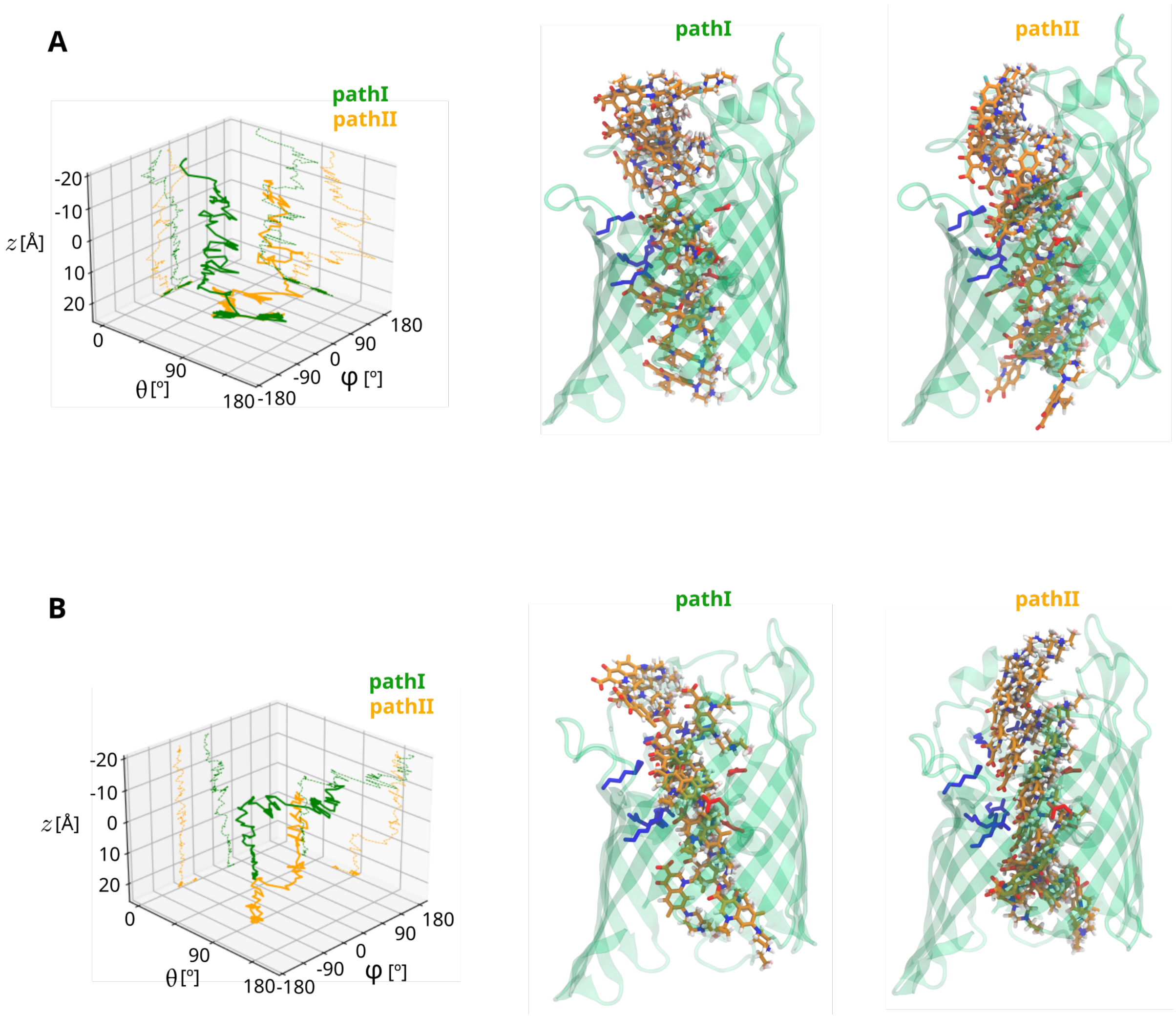

Figure S4: The input pathways obtained from the MCPS calculations that were used for setting up TASS simulations. The trajectories for Path I and Path II for (A) OmpK35 and (B) OmpE35 are depicted through a 3D plot in the $z - \theta - \phi$ space. The paths are shown as solid lines and their 2D projections as dotted lines. For both systems, the corresponding molecular configurations are shown adjacent to the 3D plots.

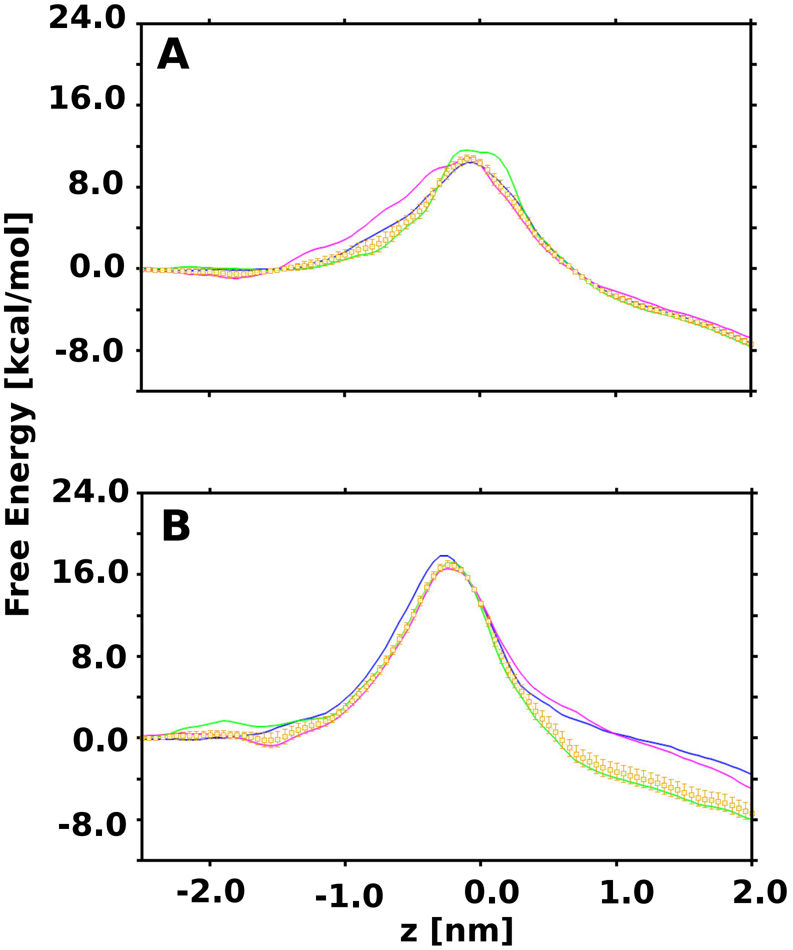

Figure S5: One-dimensional free energy estimates for enrofloxacin permeation through the (A) OmpK35 and (B) OmpE35 channels. The calculations were performed using configurations from MCPS-derived paths as inputs for the TASS simulations. Both simulations were performed with a bias on the three monomers in separate simulation runs. The average values and the associated errors were calculated using Equation 1 shown in the method section and are depicted in yellow.

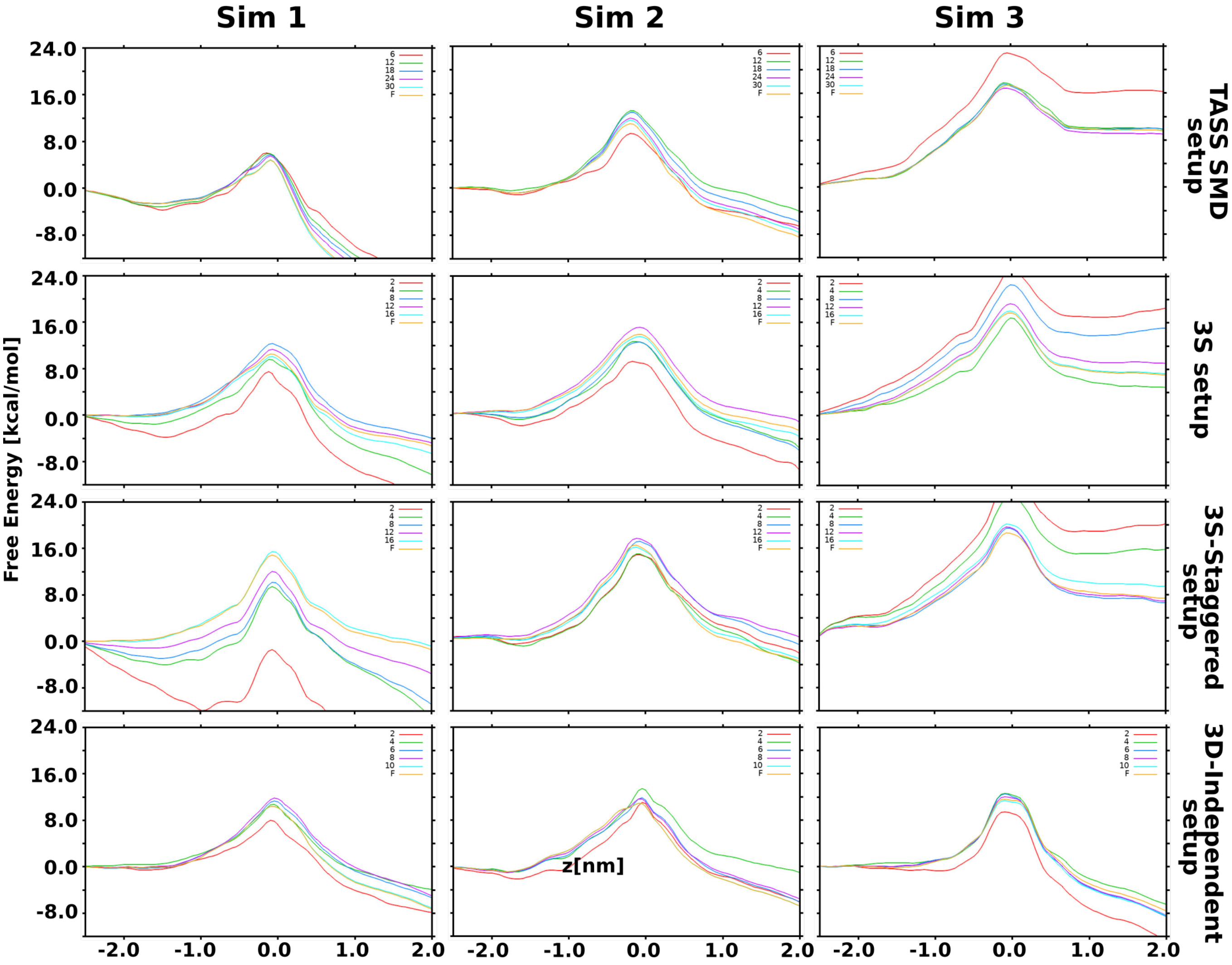

Figure S6: Convergence of 1D free energy estimates for enrofloxacin permeation through OmpK35 channel. The results obtained from TASS simulations using SMD and the three MCPS setups tested in this work are shown.

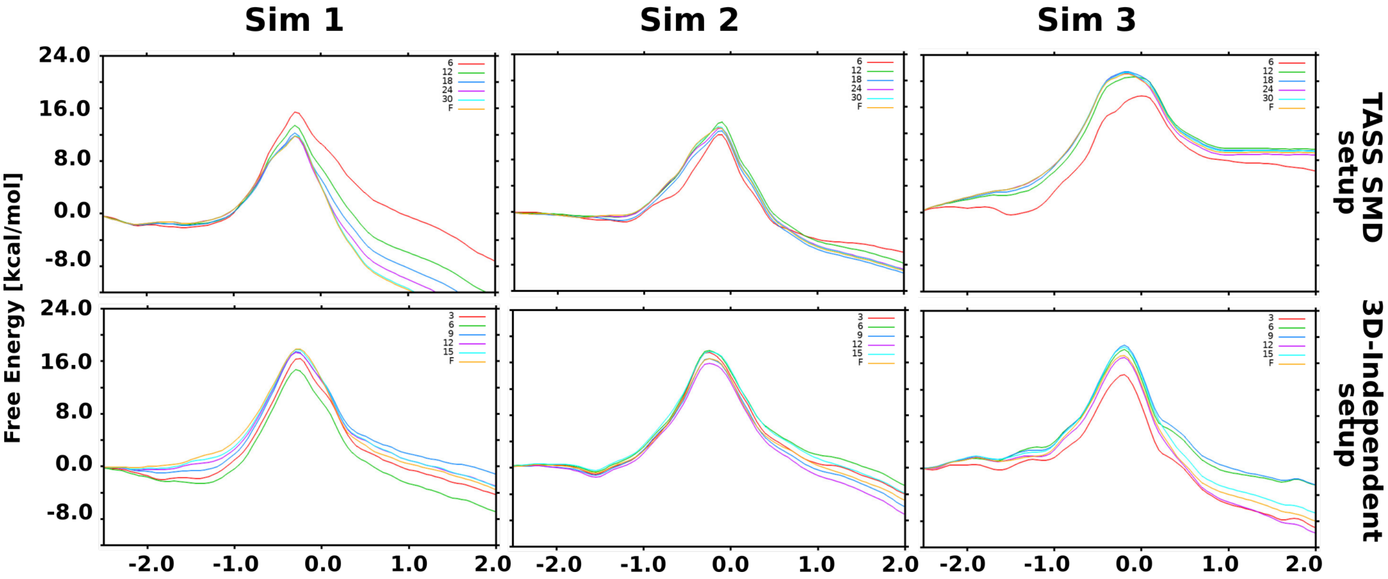

Figure S7: Convergence of 1D free energy estimates for enrofloxacin permeation through OmpE35 channel. The results obtained from TASS simulations using SMD and the 3D-Independent MCPS setup are shown.
